# Involvement of Astrocytes and microRNA Dysregulation in Neurodegenerative Diseases: From Pathogenesis to Therapeutic Potential

**DOI:** 10.3389/fnmol.2021.556215

**Published:** 2021-03-17

**Authors:** Yang Bai, Xing Su, Lianhua Piao, Zheng Jin, Rihua Jin

**Affiliations:** ^1^Department of Neurosurgery, The First Hospital of Jilin University, Changchun, China; ^2^Department of Clinical Laboratory, The Second Hospital of Jilin University, Changchun, China; ^3^College of Basic Medical Sciences, Jilin University, Changchun, China

**Keywords:** astrocytes, neurodegenerative diseases, miRNAs, biomarkers, treatment

## Abstract

Astrocytes are the most widely distributed and abundant glial cells in the central nervous system (CNS). Neurodegenerative diseases (NDDs) are a class of diseases with a slow onset, progressive progression, and poor prognosis. Common clinical NDDs include Alzheimer’s disease (AD), Parkinson’s disease (PD), amyotrophic lateral sclerosis (ALS), and Huntington’s disease (HD). Although these diseases have different etiologies, they are all associated with neuronal loss and pathological dysfunction. Accumulating evidence indicates that neurotransmitters, neurotrophic factors, and toxic metabolites that are produced and released by activated astrocytes affect and regulate the function of neurons at the receptor, ion channel, antigen transfer, and gene transcription levels in the pathogenesis of NDDs. MicroRNAs (miRNAs) are a group of small non-coding RNAs that play a wide range of biological roles by regulating the transcription and post-transcriptional translation of target mRNAs to induce target gene expression and silencing. Recent studies have shown that miRNAs participate in the pathogenesis of NDDs by regulating astrocyte function through different mechanisms and may be potential targets for the treatment of NDDs. Here, we review studies of the role of astrocytes in the pathogenesis of NDDs and discuss possible mechanisms of miRNAs in the regulation of astrocyte function, suggesting that miRNAs may be targeted as a novel approach for the treatment of NDDs.

## Introduction

Neurodegenerative diseases (NDDs) refer to slowly progressive neurological diseases that are caused by neuronal degeneration and apoptosis (Heemels, 2016). Clinically, NDDs mainly include Alzheimer’s disease (AD), Parkinson’s disease (PD), Huntington’s disease (HD), and amyotrophic lateral sclerosis (ALS). Neurodegenerative diseases mainly manifest as motor and cognitive dysfunction (Giovagnoli et al., 2018; Ruppert et al., 2020). Histopathological studies of these diseases have shown that neurons slowly and gradually become apoptotic, exhibit abnormal protein aggregation, and form inclusion bodies, suggesting that they may have similar disease progression mechanisms (Lau et al., 2020).

Astrocytes are widely distributed glial cells in the central nervous system (CNS), the abundance of which is 10- to 50-times higher than neurons (Ridet et al., 1997). Astrocytes confer the basic skeleton of the CNS and participate in its functional activity (Khakh and Sofroniew, 2015). The main functions of astrocytes include: (i) the synthesis and secretion of various cytokines, playing an important role in the survival, development, and differentiation of neurons (Sofroniew, 2014); (ii) support, isolation, and insulation (Liddelow and Barres, 2017); (iii) the regulation of neuronal metabolism and stabilization of the microenvironment, transporting neuroactive amino acids, participating in glucose metabolism, regulating intracellular calcium levels, and buffering potassium ions (Sofroniew and Vinters, 2010); (iv) the synthesis and secretion of neurotrophic factors, chemokines, and cytokines that participate in immune responses to neurological diseases (Brambilla, 2019); (v) the promotion of nerve regeneration and repair (Anderson et al., 2016); (vi) participation in the migration of axons and neuroblasts (Seyedhassantehrani et al., 2016); (vii) antioxidant damage; and (viii) interactions with neurons in information exchange and neurotransmitter transfer (Bachoo et al., 2004). Increases in calcium concentrations in astrocytes enhance excitability and play an important role in synapses, the number and efficiency of synapses, the formation of neural nuclei, and the development of NDDs (Lind et al., 2018). Astrocytes also participate in formation of the blood-brain barrier (BBB). The BBB is composed of endothelial cells, a basement membrane, astrocyte foot processes, microglia, and an extracellular matrix. The dysfunction of astrocytes, the BBB, and neurons plays an important role in NDDs.

Numerous studies in recent years have shown that microRNAs (miRNAs) are abnormally expressed in neurons during the pathogenesis of NDDs (Goodall et al., 2013). miRNAs are involved in the pathogenesis of NDDs by directly or indirectly regulating the function of astrocytes. miRNAs have a relatively small molecular weight. They can readily pass the BBB and exhibit stable expression in peripheral blood (Leidinger et al., 2013), which can be isolated and examined by standard laboratory techniques. miRNAs have been widely studied concerning the diagnosis and pathogenesis of NDDs. Here, we review recent studies of the role of astrocytes in the pathogenesis of NDDs and discuss possible mechanisms of miRNAs in regulating astrocyte function, suggesting that miRNAs can be targeted as a novel approach for the treatment of NDDs.

## Pathological Functions of Astrocytes in Neurodegenerative Diseases

The activation of astrocytes is a main pathological feature of NDDs and an adaptive defense response that provides essential metabolic support for neurons. The formation of free radicals, inflammation, elevations of the excitatory neurotransmitter glutamate, and neuronal degeneration that are caused by the dysfunction of astrocyte activation are closely related to the development of NDDs (Liddelow et al., 2017; Rivetti Di Val Cervo et al., 2017; Neal and Richardson, 2018; Yamanaka and Komine, 2018). Astrocytes are diverse types of glial cells with complex and diverse structures and functions. Accumulating evidence indicates that astrocytes present differences in morphological structure, molecular expression, basic function, and stress response. The activation of astrocytes in NDDs is a complex and multifaceted process. During this process, astrocyte-related gene and protein expression and the morphological structure and physiological function of astrocytes undergo progressive changes. The absence of the original function of astrocytes or an increase in the toxic function of astrocytes can cause nerve excitotoxicity, oxidative stress, and inflammatory response in NDD development (Acosta et al., 2017; Neal et al., 2018; Kaur et al., 2019). Also, recent studies have shown that abnormal or degenerative astrocytes can severely disrupt the transmission of information between astrocytes and neurons, resulting in neuronal dysfunction and NDDs (Turnquist et al., 2016; Gómez-Gonzalo et al., 2017; Bussian et al., 2018; Di Domenico et al., 2019). Therefore, astrocytes can play a crucial role in the pathogenesis of NDDs (Table 1).

**Table 1:** Summary of astrocyte dysfunction in neurodegenerative disease.

Diseases	Signaling molecules/pathways/functions	References
Alzheimer’s disease	Inflammatory factors can induce astrocyte to produce Aβ Activated astrocyte promote Aβ production and inhibit Aβ degradation Pathological accumulation of tau protein Release of inflammatory cytokines to accelerate the formation of neurofibrillary tangles	Blasko et al. (2000) Yang et al. (2016) Yang (2019) Birch et al. (2014)
Parkinson’s disease	Release of inflammatory factors (TNF-α, IL-6, NO, IL-1β) Astrocytes participate in oxidative stress and excitotoxicity of Parkinson’s disease Express protein (S100β) participates in the pathogenesis of Parkinson’s disease	Lee et al. (2016) Morale et al. (2006) Muramatsu et al. (2003)
Huntington’s disease	Decrease Kir4.1 K^+^ channel functional expressio Impair Ca^2+^ and glutamate signals Alter glutamate signal and induce neuronal excitotoxicity	Tong et al. (2014) Jiang et al. (2016) Bradford et al. (2009)
Amyotrophic lateral sclerosis	Glutamate transport dysfunction Inhibitory effect of GluR2 on calcium permeation Lead to mitochondrial dysfunction Inflammatory factors activate caspase-3 apoptosis Toxic effect of SOD1 on motor neuron Oxidative stress leads to nutritional factors deficiency and SOD1 mutation	Jordan et al. (2018) Vermeiren et al. (2006) Kosuge et al. (2018) Kalmar et al. (2014) Krishnan et al. (2008) Gong et al. (2000)

## Astrocytes and Alzheimer’S Disease

The inflammatory response in the brain in AD patients involves the activation of microglia and astrocytes, morphological changes in microglia, and increases in the number, volume, and activity of astrocytes (Meraz-Ríos et al., 2013). Activated astrocytes can also cause neuropathological changes through the expression of a large number of inflammatory factors. The expression of cytokines [e.g., interleukin-1β (IL-1β), IL-6, tumor necrosis factor-β (TNF-α), and transforming growth factor-β (TGF-β)] is upregulated before amyloid-β (Aβ) aggregation and tau protein hyperphosphorylation, indicating that neuroinflammation is involved in early stages of AD (Schuitemaker et al., 2009). Inflammatory factors can also activate astrocytes to produce Aβ. For example, interferon-γ (IFN-γ), together with TNF-α and IL-1β, can stimulate astrocytes to produce a large number of Aβ_1–40_ and Aβ_1–42_ proteins (Blasko et al., 2000). Amyloid β, in turn, activates astrocytes to release more cytokines. For example, Aβ has been shown to increase TNF-α levels, which is considered a factor that is involved in AD-related cognitive impairment (Veeraraghavalu et al., 2014; Yang et al., 2016). In the brain in AD patients, reactive astrocytes were reported to overexpress β-secretase 1 (BACE-1) mRNA and protein levels and promote the production of Aβ (Rossner et al., 2005). In AD, activated astrocytes can form halos that surround neuritic plaques. The formation of Aβ is closely related to activated astrocytes (Yang, 2019). Additionally, astrocytes participate in Aβ uptake and clearance from brain parenchyma to the perivascular space through the BBB. Astrocyte dysfunction leads to a decrease in Aβ uptake and clearance (Rolyan et al., 2011). *In vitro*, Aβ can form a complex with apolipoprotein E or serum amyloid P-complement C1q (SAP-C1q) and upregulate the expression of neprilysin (NEP) and scavenger receptor B family member 1 (SCARB1) in normal astrocytes, thereby inducing the further uptake of Aβ through SCARB1 and degradation of Aβ by NEP. However, astrocytes from AD patients did not exhibit increases in NEP or SCARB1 gene expression in response to Aβ–apolipoprotein E or Aβ-SAP–C1q complexes. These results indicated that astrocyte function is altered in AD (Mulder et al., 2012). Moreover, astrocytes can be activated in the AD brain to promote an inflammatory cascade, and neuroinflammation, in turn, promotes tau lesions and causes the pathological accumulation of tau protein (Birch et al., 2014). In conclusion, Aβ and many pro-inflammatory cytokines can activate astrocytes. Reactive astrocytes can release nitric oxide (NO), IL-1, IL-6, and other proinflammatory cytokines, thereby accelerating the formation of neurofibrillary tangles (NFTs; Allaman et al., 2011).

## Astrocytes and Parkinson’S Disease

Parkinson’s disease is a kind of NDD that is characterized by the progressive loss of dopaminergic neurons in the substantia nigra of the midbrain and the formation of Lewy bodies (Zhang et al., 2017). Many previous studies explored the etiology and pathogenesis of PD from the perspective of dopaminergic neurons, but researchers have begun to realize that other important and often neglected phenomena contribute to pathological changes in the brain in PD, including the proliferation and activation of glial cells (Cacace et al., 2017). In patients with PD and animal models of PD, the substantial loss of dopaminergic neurons occurs, in addition to moderate astrocyte activation in the dense part of the substantia nigra (Kuter et al., 2018). Astrocyte activation can produce TNF-α, IL-1β, NO, prostaglandin-E2 (PGE-2), and other inflammatory factors. These proinflammatory factors can initiate the activation of signal transduction pathways in dopaminergic neurons in different ways, eventually leading to dopaminergic neuron loss (Lee et al., 2016). TNF-α binds to specific TNF-α receptors on dopaminergic neurons and activates the proapoptotic pathway. Studies have shown that TNF-α receptor activation can cause dopaminergic neurons to degenerate through activation of the intracellular C_2_ ceramide pathway (Hunot et al., 1997; Qin et al., 2007). The IL-1β expression is extremely low in the normal CNS, and its expression is significantly upregulated in many inflammatory and degenerative diseases. IL-1β can influence the function of astrocytes, interfere with signal transmission between neurons and glial cells, and promote the pathogenesis of PD (Saghazadeh et al., 2016). Lipopolysaccharide and inflammatory factors can activate astrocytes to express inducible nitric oxide synthase (iNOS) and promote NO release (Ko et al., 2018). When astrocytes are activated by a combination of cytokines, including TNF-α, IL-1, and TNF-γ, the stable astrocyte becomes highly vulnerable to apoptosis and may cause neuronal damage or cell death by releasing free radicals and glutamate (Choi et al., 1999). Astrocytes are the main detoxification site of 1-methyl-4-phenyl-1,2,3,6-tetrahydropyridine (MPTP), which is metabolized to 1-methyl-4-phenylpyridinium (MPP^+^) by monoamine oxidase B. Once entered in dopaminergic neurons in the substantia nigra, MPP^+^ inhibits mitochondrial complex 1 and leads to oxidative stress, the loss of adenosine triphosphate, protein nitration, and the apoptosis of dopaminergic neuron (Morale et al., 2006). S100β is a Ca^2+^-binding protein that is highly expressed in injured brain tissue. Muramatsu et al. examined the expression of S100β in the striatum, substantia nigra neurons, and glial cells in mice that were subjected to MPTP-induced dopaminergic neuron injury and observed S100β-positive staining only in GFAP^+^ astrocytes, indicating that astrocytes are activated in MPTP-induced dopaminergic neuron injury and may contribute to the pathogenesis of PD (Muramatsu et al., 2003). In summary, activated astrocytes may play different roles in the pathogenesis of PD, which requires further study to identify their role in NDDs.

## Astrocytes and Huntington’S Disease

Huntington’s disease is a progressive NDD that can lead to motor, cognitive, and mental disorders. Cytosine-adenine-guanine (CAG) repeat expansions in the *HTT* gene lead to the formation of mutant huntingtin (mHTT; Hsiao et al., 2013). The nuclear aggregation of mHTT protein is the main cause of degeneration, inflammation, apoptosis, and neuronal loss in the cortex and striatum (Zhang et al., 2018). Astrocytes are more efficient than neurons at clearing mHTT aggregates, so astrocytes are more resistant to mHTT aggregation (Zhao et al., 2016). However, mHTT aggregation in astrocytes can alter glutamate signaling and cause neuronal excitotoxicity (Shin et al., 2005). Medium spiny neuron (MSN) dysfunction in the striatum is a characteristic change in HD. Tong et al. (2014) found that alterations of striatal MSN excitability in HD may be caused by disturbances of astrocyte-mediated K^+^ homeostasis. In a mouse model of HD, the frequency, duration, and amplitude of spontaneous Ca^2+^ signals were significantly reduced. The dysfunction of Ca^2+^ and glutamate signaling could be largely rescued by the astrocyte-specific restoration of Kir4.1, suggesting the important contribution of homeostatic K^+^ mechanisms that are known to be lower in a mouse model of HD (Jiang et al., 2016). These dysfunctions lead to a reactive state of astrocytes and suggest that neurotoxicity that causes inflammation might be a secondary effect of HD. In the inflammatory state, microglia activate astrocytes to release TNF-β and IL-1β, decreasing synaptic maintenance and phagocytic activity and increasing the degeneration of neurons and oligodendrocytes (Bradford et al., 2009; Liddelow et al., 2017).

## Astrocytes and Amyotrophic Lateral Sclerosis

Amyotrophic lateral sclerosis is a chronic progressive NDD that selectively invades anterior horn cells in the spinal cord, motor neurons in the brainstem, and motor cortex (Owens, 2017). Various pathogenic factors have been proved to be involved in the pathogenesis of ALS, including glutamate excitotoxicity, copper/zinc superoxide dismutase (*SOD1*) gene mutations, mitochondrial dysfunction, oxidative stress, glial cell dysfunction, cytoskeleton disorders, abnormal protein folding and aggregation, nutritional factor deficiencies, and inflammation (Kiernan et al., 2011; Lyon et al., 2019).

In addition to the aforementioned mechanisms involved in the pathogenesis of ALS, recent studies have also focused on the role of astrocytes. In ALS, motor neuron protein aggregation is a hallmark of the disease. Tripathi et al. showed that reactive astrocytes increased axonal and cytoplasmic protein inclusions by releasing transforming growth factor β1, which disrupted motor neuron autophagy through the mammalian/mechanistic target of rapamycin (mTOR) pathway (Tripathi et al., 2017).

Glutamate transport dysfunction in astrocytes is the main cause of higher extracellular glutamate levels. Increases in glutamate levels were found in cerebrospinal fluid in ALS patients. The inhibition of glutamate uptake can induce the selective degeneration of motor neurons. Glutamate transporter levels in astrocytes were downregulated in lesions of the spinal cord and motor cortex. Rothstein et al. (1995) developed C-terminal, antioligopeptide antibodies that were specific for each glutamate transporter. Excitatory amino acid transporters were selective for neurons, while glutamate transporter 1 was selective for astrocytes. They showed that the expression of glutamate transporter 1 with antisense oligonucleotides caused a dramatic elevation of extracellular glutamate and neuronal injury in specific regions of the motor cortex and spinal cord, suggesting that this change was associated with motor neuron injury (Rothstein et al., 1995; Heath and Shaw, 2002; Jordan et al., 2018). Astrocytes regulate the expression of glutamate receptor 2 (GluR2) subunits and the susceptibility of motor neurons to excitotoxicity.

Amyotrophic lateral sclerosis is associated with the expression of SOD1 mutations in astrocytes and inhibition of the ability of GluR2 to regulate Ca^2+^ ion penetration, thus making motor neurons more sensitive to excitotoxicity and suggesting that astrocytes affect motor neuron function (Vermeiren et al., 2006; Van Damme et al., 2007). Reactive astrocytes can damage mitochondria in co-cultured neurons (Barbeito et al., 2004). Studies have reported intracellular mitochondrial dysfunction in the pathogenesis of ALS and alterations of calcium homeostasis. A reduction of Ca^2+^ ions can affect neuronal transmission (Kawamata and Manfredi, 2010). Neuroinflammation plays an important role in the pathogenesis of ALS.

In recent years, inflammation in the spinal cord has received widespread attention as important pathogenesis of ALS. PGE-2, a key pro-inflammatory mediator, is elevated in serum and CSF of patients with sporadic ALS and G93A mice (Iłzecka, 2003; Miyagishi et al., 2017). Also, the expression of cyclooxygenase-2 (COX-2), the key enzyme for the arachidonic acid synthesis of prostaglandins, is increased in the spinal cord of ALS patients and model mice (Yasojima et al., 2001; Kosuge et al., 2018). Such cytokines as TNF-α and Fas L are secreted, which then activate caspase-8 and regulate nuclear factor-κB (NF-κB), inflammatory cytokines, chemokines, and oxygen free radicals to cause an inflammatory response in glial cells, activate the classic caspase-3 apoptotic pathway, and affect neuronal function (Kalmar et al., 2014).

Astrocytes that express SOD1 mutations have a significant toxic effect on neurons (Krishnan et al., 2008). In transgenic mouse models of ALS, SOD1 mutations promoted cell death, and ALS neurons that expressed a SOD1 mutation were vulnerable to damage. Reductions of SOD1 expression in neurons delayed the occurrence of ALS and prolonged the life of transgenic mice (Gong et al., 2000). The pathogenesis of ALS is not the result of a single factor but rather a variety of cellular interactions. The regulation of astrocyte function, improvements in the regeneration environment of motor neurons, and promotion of the survival and regeneration of motor neurons are new research avenues that are being studied for the treatment of ALS.

## Involvement of miRNAs in Astrocyte Dysfunction in Neurodegenerative Diseases

**Figure 1:**
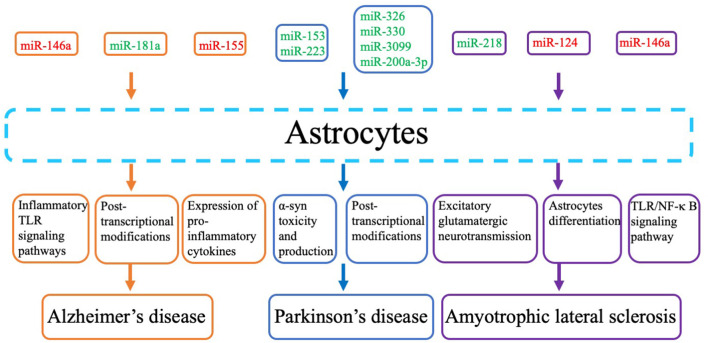
Pathogenic mechanisms and pathways and related microRNAs (miRNAs) that are considered key molecular players in the contribution of astrocytes tossssss neurodegenerative diseases. Red miRs indicate upregulated miRNAs, and green miRs indicate downregulated miRs.

microRNAs (miRNAs) are a class of endogenous non-coding single-stranded, ~22-nucleotide RNA molecules, which regulate gene expression post-transcriptionally by base-pairing with their target mRNAs (Stenvang and Kauppinen, 2008). They play an important role in cell fate determination, cell differentiation, organ development, and physiology and are also involved in various pathologies in humans (Almeida et al., 2011; Desvignes et al., 2015). miRNAs regulate target gene mRNA mainly by cutting off the target gene’s RNA molecule, thereby inhibiting target gene translation and inhibiting recombination (Han et al., 2019). By binding to the RNA-induced silencing complex, the nucleotide sequence of miRNA allows targeted base pairing with the 3′-untranslated region of complementary mRNA. miRNA sequence-specific mRNA silencing can be achieved through two mechanisms: (i) if the miRNA-mRNA sequence is sufficiently complementary, then transcriptional cleavage will occur; and (ii) if there is a lack of complementarity but there are still some remaining complementary miRNA sites on the mRNA, then translation suppression can be achieved (Zeng et al., 2003; Bruen et al., 2019). Emerging studies suggest that many miRNAs are expressed in astrocytes, and some of them have been shown to participate in astrocyte differentiation, activation, and specification of the inflammatory response (Neal and Richardson, 2018). Neo et al. found that miR-124 increased astrocyte differentiation by directly downregulating the expression of histone-lysine *N*-methyltransferase (Ezh2; Neo et al., 2014). Meares et al. (2018) reported that miR-31 promoted astrocyte development and specification by reducing the levels of Lin28, a stem cell factor that is implicated in neural precursor cell renewal. Van Scheppingen et al. (2018) reported that the miR-146a and miR-147b were differentially expressed in temporal lobe epilepsy patients with hippocampal sclerosis, and were related to increased expression of genes associated with the inflammatory response. Overexpression of miR-146a and miR-147b could reduce the expression of the pro-inflammatory mediators IL-6 and COX-2 in astrocytes and decrease the proliferation of astrocytes (Van Scheppingen et al., 2018), suggesting that these two miRNAs may be potential therapeutic targets for the treatment of neurological disorders that are associated with inflammation. To present, many studies have suggested that miRNAs are also involved in astrocyte dysfunction in NDDs (Figure 1, Table 2).

**Table 2:** Important miRNAs and their targets that are involved in regulating astrocyte during neurodegenerative diseases (NDDs).

NDDs	MicroRNA implicated	Targets (mRNA)	Involved in (signaling) pathways	References
AD				
	miR-146a	IRAK-1, IRAK-2	Inflammatory TLR signaling pathways	Cui et al. (2010)
	miR-181a	GLT-1	Post-transcriptional modifications	Zumkehr et al. (2018)
	miR-155	SOCS1	Expression of pro-inflammatory cytokines	Guedes et al. (2014)
PD				
	miR-153, miR-223	HO-1	α-syn toxicity and production	Cressatti et al. (2019)
	miR-326, miR-330 and miR-3099	PINK1	post-transcriptional modifications	Choi et al. (2016)
	miR-200a-3p	MKK4	Post-transcriptional modifications	Shakespear et al. (2020)
ALS				
	miR-218	EAAT2	Excitatory glutamatergic neurotransmission	Ferraiuolo and Shaw (2018) and Ridler (2018)
	miR-124	Sox2, Sox9	Astrocytes differentiation	Zhou et al. (2018)
	miR-146a	IRAK1, TRAF6	TLR/NF-kB signaling pathway	Gomes et al. (2019)

*AD: Alzheimer’s disease; ALS: amyotrophic lateral sclerosis; NDDs: neurodegenerative diseases; PD: Parkinson’s disease*.

## Involvement of miRNAs in Astrocyte Dysfunction in AD

Extracellular Aβ plaques and intracellular Tau protein aggregates are pathological features of AD, accompanied by the loss of synapses, the death of neurons, and a chronic inflammatory response in astrocytes (Zhu and Wang, 2015; Tobore, 2019). miR-146a is located on chromosome 5 of the human genome and is widely expressed in the immune system. It is usually expressed at a low level in immune precursor cells, but its expression is upregulated as immune cells mature and activate (Starczynowski et al., 2011). miR-146a was reported to downregulate interleukin-1 receptor-associated kinase-1 (IRAK-1) in endotoxin- and cytokine-challenged human monocytes (Taganov et al., 2006) and specifically associated with the upregulation of inflammatory signaling in prion-induced neurodegeneration and temporal lobe epilepsy (Cui et al., 2010). Cui et al. (2010) found miR-146a was upregulated in human astrocytes that were exposed to Aβ. miR-146a-mediated down-regulation of IRAK-1 coupled to an NF-κB-induced up-regulation of IRAK-2 expression drove an extensively sustained inflammatory response. The interactive signaling of NF-κB and miR-146a further illustrated the interplay between inducible transcription factors and pro-inflammatory miRNAs that regulate brain IRAK expression. Therefore, the combined use of NF-κB inhibitors with miR-146a or antisense miR-146a may be a bi-pronged treatment strategy directed against IRAK-2 driven pathogenic signals (Cui et al., 2010).

Glutamate release, reuptake, and recycling are tightly regulated by neurons and astrocytes at tripartite synapses. Glutamate overload can trigger neuronal and synaptic loss, which potentially contributes to the pathogenesis of AD (Marttinen et al., 2018). Glial glutamate transporter 1 (GLT-1) contributes to the clearance and regulation of glutamate at synaptic clefts. An increase in miR-181a downregulated some synaptic proteins that are involved in plasticity in a 3xTg-AD mouse model and had a negative regulatory relationship with GLT-1, suggesting that miR-181a is a key mediator of the plasticity of glutamatergic synapses by controlling the expression of synaptic proteins, including GLT-1, in astrocytes (Zumkehr et al., 2015, 2018).

miR-155 has a wide range of functions. It is involved in many biological processes, such as the development and differentiation of hematopoietic cells and immune cells. It also participates in inflammatory reactions, the immune response, muscle development, and adipose differentiation. Higher levels of miR-155 in astrocytes were shown to induce the prolonged-expression of proinflammatory cytokines by targeting SOCS1, a negative regulator of the inflammatory gene response, in Aβ-treated astrocytes (Guedes et al., 2014). These data showed that the pathogenesis of AD is characterized by distinct neuroinflammatory events that involve the dysregulation of miRNA expression in astrocytes.

## Involvement of miRNAs in Astrocyte Dysfunction in PD

Parkinson’s disease is related to various pathological factors, including α-synuclein toxicity and other mechanisms. Cressatti et al. found the serum concentrations of miR-153 and miR-223 progressively decreased in the wild-type (WT) and GFAP.HMOX1 mice. Also, circulating levels of both miRNAs were lower in the transgenic mice compared to WT controls, while α-synuclein protein concentrations were elevated in GFAP.HMOX1 mice, relative to WT values, both of these miRNAs negatively regulated α-synuclein in the basal ganglia in GFAP.HMOX1 mice. Moreover, the overexpression of heme oxygenase-1 (HO-1) in stressed astrocytes was observed in the substantia nigra in idiopathic PD, which promoted α-synuclein toxicity and production by downregulating miR-153 or miR-223 in the CNS (Cressatti et al., 2019).

miR-326, miR-330, and miR-3099 were reported to be associated with astroglioma. The expression of these three miRNAs increased during brain development and neural stem cell (NSC) differentiation and was significantly lower in the PTEN-induced putative kinase 1 (PINK1)-knockout mouse brains. Choi et al. (2016) found that three astrocyte-related miRNAs (miR-326, miR-330, and miR-3099) increased NSC differentiation into astrocytes and were significantly downregulated in the absence of the PD gene phosphatidylinositol 3,4,5-trisphosphate 3-phosphatase and dual-specificity protein phosphatase PINK1 (Choi et al., 2016).

MPP^+^ is a well-known neurotoxin that is used to induce cell death *in vitro* in models of PD. miR-200a-3p was reported to be downregulated in MPP^+^-stimulated astrocytes. Mitogen-activated kinase- kinase 4 (MKK4) is involved in the mechanism of PD-related cell death (Saporito et al., 2000). Ogura et al. demonstrated that MKK4 inhibition could rescue cells from cell death, indicating that MKK4 played an important role in MPP^+^-induced cell death and possibly in PD pathogenesis (Ogura et al., 2019). miR-200a-3p targeted MKK4 by binding to two independent sites on the 3’-untranslated region of dual-specificity MKK4 (Map2k4)/MKK4 mRNA. Shakespear et al. (2020) reported that treatment with a miR-200a-3p mimetic attenuated cell death in MPP^+^-treated SH-SY5Y cells by suppressing both MKK4 mRNA and protein expression.

## Involvement of miRNAs in Astrocyte Dysfunction in ALS

One pathological feature of ALS in the brain is an increase in reactive astrocytes, which is associated with a low clearance efficiency of toxic excitatory glutamate neurotransmitters and impairments in neurotrophic factor secretion (Lasiene and Yamanaka, 2011). miR-218 is a motor neuron-specific miRNA that is involved in astrocyte function and the pathophysiology of ALS. miR-218 was shown to be released by dying motor neurons and taken up by astrocytes. Furthermore, miR-218 was shown to regulate the expression of the glutamate reuptake transporter excitatory amino acid transporter 2 (EAAT2) and be important for the proper regulation of excitatory glutamatergic neurotransmission (Ferraiuolo and Shaw, 2018; Ridler, 2018). Hoye et al. (2018) found that the downregulation of miR-218 ameliorated astrocyte dysfunction in ALS.

The expression of miR-124 is higher in human brain tissue, accounting for 25–48% of total miRNA in the adult brain, with the highest content in the cerebral cortex (Mishima et al., 2007). miR-124 can be combined with Jagged2 (Jag2, a ligand of Notch1) to significantly downregulate Jag2 expression, thereby inhibiting the Notch1 signaling pathway and promoting the transformation of cortical radial glial cells to astrocytes (Zhang et al., 2015). Approximately 20% of familial ALS cases are caused by SOD1 mutations (Rosen et al., 1993). Zhou et al. (2018) found that miR-124-positive cells were located mainly at sites of neurodegeneration in the spinal cord and brain stem in a transgenic mouse model of ALS. The levels of miR-124 increased in young (95-day-old) G93A-SOD1 mutant mice but decreased in older (108- and 122-day-old) mice compared with wild-type mice. These authors concluded that miR-124 is related to the differentiation of neural stem cells into astrocytes by targeting Sox2 and Sox9 (Zhou et al., 2018).

miR-146a is widely expressed in the immune system and is involved in the differentiation, proliferation, and activation of various immune cells. miR-146a was upregulated in IL-1β-stimulated human astrocytes, indicating that the effects of this miRNA are associated with the regulation of an astrocyte-mediated inflammatory response (Iyer et al., 2012). miR-146a downregulation was reported to upregulate both interleukin-1 receptor-associated kinase 1 (IRAK1) and TNF receptor-associated factor 6 (TRAF6) in murine SOD1 astrocytes by impacting the TLR/NF-κB signaling pathways, thus contributing to neuroinflammation (Gomes et al., 2019).

## Developing miRNA-Based Astrocyte-Regulatory Therapeutics to Treat Neurodegenerative Diseases

The possibility of miRNA-based strategies to treat NDDs is an exciting approach. miRNA inhibition could pair mature miRNAs and prevent binding to target genes through chemically modified oligonucleotide sequences. miRNA removes target miRNAs from cultured cells through a “sponge” or “eraser” action (Ebert et al., 2007; Van Rooij et al., 2008). miRNA mimetics or overexpression vectors have been used to rescue the suppression of endogenous miRNA expression levels in cells. With improvements in miRNA delivery, viral and non-viral vectors have been developed. Effective virus-based vectors have been developed for the stable and sustained expression of miRNAs. An intracranial injection of adeno-associated virus (AAV)-expressing miR-124–3p in APP/PS1-AD mice significantly reduced Aβ deposition and improved cognitive function in AD mice (Zhou et al., 2019), suggesting high efficiency of the virus delivery system that can deliver miRNAs to the effective target cells. Liposomes, polymer-based systems, and inorganic nanoparticles are non-viral vector delivery systems that have also been widely used in clinical research (Labatut and Mattheolabakis, 2018). The use of tissue-specific promoters may allow miRNA-mimicking vectors to have greater specificity (To et al., 2020). miRNA-124-loaded polymeric nanoparticles that were injected in the ventricles in a mouse model of PD enhanced the migration of new neurons to the lesioned striatum, culminating in improvements in motor function (Saraiva et al., 2016). Although there is no conclusive evidence that miRNA therapeutics are effective in clinical trials for NDDs, further progress in delivering miRNA therapeutics to desired sites of action may contribute to clinical development. This transgenic approach has become a promising tool for detecting the function of miRNAs in different cell types and has the potential to become a future treatment (Karthikeyan et al., 2016).

Considering the large number of studies that suggest that astrocytes are involved in the pathogenesis of NDDs through various mechanisms, various studies have investigated therapeutic strategies for NDDs by regulating astrocyte function through miRNAs. Recent evidence suggests that peroxisome proliferator-activated receptors (PPARs) are actively involved in the modulation of astrocyte metabolism in NDDs (Martin et al., 2013; Joardar et al., 2015). The activation of PPARβ/δ suppressed the CpG island hypermethylation-associated silencing of miR-181a and protected against endoplasmic reticulum stress-induced damage to astrocytes, revealing a promising target for regulating endoplasmic reticulum stress-induced astrocyte injury (Pilakka-Kanthikeel et al., 2015). Ghasemi-Kasman et al. (2018b) used miR-302/367 and sodium valproate to demonstrate the possibility of astrocytes transforming into oligodendrocyte progenitor and myelin cells in a cuprizone-induced demyelination model. It was suggested that the specific targeting of astrocytes *in vivo* for forced expression of miR-302/367 cluster could increase the ability to repair myelin insults in cerebral structures through the generation of oligodendroglia by astrocytes, which opened a possible new avenue to enhance cerebral repair in NDDs (Ghasemi-Kasman et al., 2018b). The *in vivo* reprogramming of reactive astrocytes into functional neurons also suggested a new treatment strategy for AD. Ghasemi-Kasman et al. further found that the expression of a miR-302/367 cluster by the application of lentiviral particles enhanced the ability of the hippocampus and other brain structures to regenerate following neuronal loss in mice (Ghasemi-Kasman et al., 2017). These researchers also investigated the possible contribution of miR-302/367-induced induced neuronal activation to behavioral improvement and neural repair in an animal model of AD. Lentiviral particles assembled miR-302/367^ +^ green fluorescent protein were injected in the dentate gyrus of the hippocampus, and short-term memory and spatial memory improved. Thus, the *in vivo* reprogramming of reactive astrocytes to neurons by the miR-302/367 cluster could be a feasible strategy to rescue learning and memory in AD patients (Ghasemi-Kasman et al., 2018a). Astrocyte toxicity contributes to motor neuron degeneration in ALS. Varcianna et al. performed a study to verify the link between miR-494-3p dysregulation and astrocyte-mediated motor neuron death. They found that miR-494-3p downregulated semaphorin 3A (Sema3A) levels in motor neurons and increased motor neuron survival *in vitro*, suggesting that miR-494-3p may be a therapeutic target for ALS (Varcianna et al., 2019).

Exosomes are 40–100 nm in diameter. They are a type of lipidic bilayer membrane-encapsulated vesicles that are released from cells to the extracellular space. Exosomes and the miRNAs, mRNAs, and proteins that they contain can reflect the pathophysiological state of source cells and are an important means of intercellular communication and the transport of biologically active substances, such as during the immune response, protein metabolism, and cell damage. The relationship between exosomal miRNAs and the pathogenesis of NDDs has prompted researchers to study their therapeutic potential. Based on *in vivo* striatal injections, exosome-mediated miRNAs (specifically miR-124a) were shown to play a role in neuron-to-astrocyte signaling. Exosomes that were isolated in a conditioned medium that contained miRNA were internalized into astrocytes and increased miR-124a levels in astrocytes and GLT1 proteins in ALS. In a mouse model of end-stage ALS, miR-124a was selectively reduced in the spinal cord. Therefore, the *in vivo* exogenous transmission of miR-124a by a stereogenic injection prevented the further pathological loss of GLT1 protein in cultured astrocytes in the ALS mouse model (Morel et al., 2013; Izadpanah et al., 2018). Exosomes that were loaded with miRNAs have broad prospects for the treatment of NDDs. However, the application of exosome manipulation techniques for the effective treatment of NDDs needs to be optimized and standardized.

Research on the application of miRNA expression technology to regulate astrocyte function for the treatment of NDDs is limited. Nonetheless, strong evidence of the role of astrocytes in the pathogenesis and progression of NDDs and advances in miRNA treatment technology may provide a theoretical basis for the early application of this therapeutic strategy in NDDs.

## Conclusions

We reviewed current knowledge of the role of astrocytes in NDDs. Several studies have focused on interactions between miRNAs and astrocyte functions to elucidate the molecular mechanisms of NDDs. The regulation of astrocyte functions in various mechanisms that contribute to NDDs may lead to new therapeutic strategies. However, the cell-specific delivery of miRNAs in the brain *in vivo* remains challenging. Manipulations of miRNAs can also have unpredictable effects on mRNA expression. Several challenges remain in the development of new therapeutic strategies that target miRNAs to regulate astrocyte function. Further research in this area will help fill gaps in our understanding of the role of miRNAs in NDDs and may have important clinical implications.

## Author Contributions

This manuscript was primarily written by YB, XS, and ZJ. The figure was produced by LP and RJ. RJ contributed to editing the manuscript. All authors contributed to the article and approved the submitted version.

## Conflict of Interest

The authors declare that the research was conducted in the absence of any commercial or financial relationships that could be construed as a potential conflict of interest.
